# Association of Physical Activity Intensity and Light/Deep Sleep in Young People from Southern Spain

**DOI:** 10.3390/children12050534

**Published:** 2025-04-22

**Authors:** Pablo Ramírez-Espejo, José Luis Solas-Martínez, Manuel J. de la Torre-Cruz, Alberto Ruiz-Ariza

**Affiliations:** 1 Department of Didactics of Musical, Plastic and Corporal Expression, University of Jaen, 23071 Jaén, Spain; pre00005@red.ujaen.es (P.R.-E.); arariza@ujaen.es (A.R.-A.); 2 Department of Psychology, University of Jaén, 23071 Jaén, Spain; majecruz@ujaen.es

**Keywords:** healthy habits, exercise, sleep, adolescent

## Abstract

**Background**: Previous research has examined the relationship between physical activity (PA) and sleep quality in young people. However, studies exploring how different intensities of PA relate to light and deep sleep, using objective measurement tools, remain scarce. Therefore, the aim of the present study was to analyze the potential associations between PA intensity and sleep stages and to determine the most effective intensity of PA for positively influencing sleep during adolescence. **Methods**: The sample consisted of 1072 Spanish pre-adolescents and adolescents (53% girls and 47% boys) aged 13.03 ± 1.79 years. Sleep and intensity of PA were measured using the Xiaomi Mi Band 4 smartband. Sleep was categorized as light or deep, while PA intensity was determined by heart rate (HR) and classified as light (resting to 50% of maximum HR), moderate (50–70% of max HR), and vigorous (70–85% of max HR). **Results**: Analyses revealed that greater daily time spent in light-intensity PA was associated with less light sleep and more deep sleep. Additionally, vigorous-intensity PA was positively associated with increased deep sleep. No negative associations were observed between moderate-intensity PA and sleep quality. In conclusion, this study suggests that promoting prolonged light-intensity activities (e.g., walking) or short bursts of vigorous activity (e.g., sports participation) may enhance sleep quality during pre-adolescence and adolescence.

## 1. Introduction

Pre-adolescence and adolescence are critical developmental stages during which sleep plays a pivotal role in overall health and functioning. Sufficient and high-quality deep sleep is associated with improved well-being, as well as enhanced cognitive and academic performance. However, recent studies have reported a shift toward later bedtimes and an alarming decline in both the duration and quality of sleep among young people [1,2]. Poor or superficial sleep has been linked to psycho-emotional impairments, including heightened levels of anxiety and depression, and to academic difficulties such as decreased attention, memory, and learning capacity [3,4,5].

Previous research has indicated that approximately half of European adolescents experience poor sleep quality, with girls being disproportionately affected [6]. A study assessing sleep quality and duration among 1717 European adolescents aged 13 to 16 from Spain, Iceland, and Estonia found that Spanish youth were particularly prone to poor sleep outcomes. Specifically, 68% of Spanish adolescents failed to achieve the recommended 8–10 h of nightly sleep, and 51% reported low sleep quality. Among them, adolescents from southern Spain (Seville, Andalusia) stood out for having the poorest sleep quality, with a higher likelihood of inadequate sleep patterns (OR = 1.22) and a higher score on the Pittsburgh Sleep Quality Index (PSQI = 5.11), compared to their northern European peers (OR = 1.07–1.40; PSQI = 4.35) [7]. These findings underscore the urgency of further research into sleep quality among this population.

Multiple sociodemographic and psychological factors affect sleep during pre-adolescence and adolescence. Beyond global disruptions such as the COVID-19 pandemic, which exacerbated sleep disturbances among adolescents [8], biological and environmental contributors are also crucial. Pubertal changes, increased academic pressure, and excessive screen time before bed have all been shown to disturb sleep patterns [9]. Additionally, adolescents experiencing fragmented or insufficient sleep are more likely to engage in risky behaviors such as bullying, substance use, and excessive internet consumption [9]. Age and gender-related differences have also been reported, with older adolescents sleeping fewer hours than younger ones [10], and girls generally exhibiting poorer sleep patterns than boys [6]. Furthermore, family dynamics and socioeconomic status significantly influence sleep quality. For example, high parental expectations and household stress have been associated with unfavorable sleep habits [3].

Taken together, these findings emphasize the essential role of sleep in regulating emotional and behavioral outcomes during pre-adolescence and adolescence. Among the modifiable lifestyle factors that impact sleep, physical activity (PA) has been identified as a key determinant. The World Health Organization underscores the interrelatedness of PA and sleep as foundational components of youth health and holistic development [11]. Regular PA enhances sleep by regulating circadian rhythms, increasing melatonin secretion, and promoting thermoregulatory mechanisms that facilitate sleep onset [12]. Moreover, PA reduces stress and anxiety, contributing to deeper and more restorative sleep [13]. In general, empirical evidence supports a positive association between PA and sleep in adolescents, with those meeting the minimum daily PA recommendations (i.e., at least 60 min of moderate-intensity PA) showing better sleep outcomes than their inactive counterparts [14,15]. Most existing studies report that consistent engagement in moderate-to-vigorous PA is associated with reduced insomnia and improved overall sleep quality in adolescent populations [13]. However, there remains limited research comparing the specific effects of PA at varying intensities, measured with objective instruments, on different sleep stages such as light and deep sleep.

Given these gaps, the aim of the present study was to analyze the associations between light (rest–50% max heart rate, HR), moderate (50–70% max HR), and vigorous (70–85% max HR) PA intensities and sleep quality during pre-adolescence and adolescence. Understanding these associations can inform educational, familial, and public health initiatives aimed at fostering active lifestyles that enhance sleep quality in young populations.

## 2. Materials and Methods

### 2.1. Participants

A total of 1072 Spanish youth (53% girls and 47% boys) from five educational centers in Andalusia, southern Spain, participated in this cross-sectional quantitative study. The students were between 10 and 16 years old (13.03 ± 1.79 years) and had a body mass index (BMI) of 20.33 ± 4.01 kg/m^2^. Statistically significant gender differences were observed in weight (*p* < 0.001), height (*p* < 0.001), maternal education level (*p* < 0.001), and deep sleep (*p* = 0.007). Participants were selected through convenience sampling from the participating schools. Table 1 presents the anthropometric and sociodemographic characteristics of the sample.

### 2.2. Measures

#### 2.2.1. Dependent Variables: Light and Deep Sleep

Light and deep sleep were assessed using the Xiaomi Mi Band 4 smartband. The validity and reliability of this device have been previously confirmed by de la Casa-Pérez et al. [16]. For the analysis, the average number of minutes per day spent in light and deep sleep was used.

#### 2.2.2. Independent Variables: Daily Physical Activity Intensity

Daily PA intensity was determined based on HR data recorded with the Xiaomi Mi Band 4 smartband. A previous study validated the use of this device for tracking daily HR data [16]. The classification criteria established by the American Heart Association [17] were applied, defining light intensity as the range between resting HR and 50% of the maximum HR; moderate intensity as 50–70% of the maximum HR; and vigorous intensity as 70–85% of the maximum HR.

#### 2.2.3. Confounding Variables

Previous studies have identified age, sex, and BMI as variables that influence both systematic PA [18] and sleep patterns in adolescents [6,10]. Therefore, these variables were considered covariates in the current study. Body weight and height were measured using an ASIMED^®^ Type B Class III digital scale (Barcelona, Spain) and a SECA^®^ 214 portable stadiometer (SECA Ltd., Hamburg, Germany). All measurements were taken with participants wearing light clothing and no footwear. The BMI was calculated as weight (kg)/height^2^ (m). Sociodemographic information was collected using a structured questionnaire, which included questions regarding participants’ age and family background.

### 2.3. Procedure

This study was presented to school directors, and informed consent was obtained from parents or legal guardians. Each participant was assigned a unique anonymous code to ensure confidentiality. Data collection was conducted between February and May 2022 during school hours allocated for research purposes. The process was divided into two main phases. In the first phase, participants’ sleep and HR data were collected over seven consecutive days using Xiaomi Mi Band 4 smartbands. Each participant received a smartband configured with their individual anthropometric data and was instructed to wear it on their left wrist continuously for nine days. Data from the first and last day were excluded (as the devices were received after the day had started and returned before the day ended). Thus, only the seven full 24 h recordings from days 2 to 8 were analyzed. This process was repeated until data from all participants had been gathered. In the second phase, sociodemographic data were gathered using structured questionnaires.

### 2.4. Statistical Analysis

Results are presented as means and standard deviations for continuous variables, and percentages for categorical variables. Independent samples *t*-tests were used to assess gender differences in continuous variables, and Chi-square tests for categorical variables. To examine the relationship between the daily time spent in PA at different intensities (light, moderate, and vigorous) and sleep duration (light and deep sleep), multiple linear regression analyses were performed. Sleep variables served as the dependent variables, while PA intensity levels were the independent variables. Age, sex, and BMI were included as covariates. All analyses were conducted using SPSS version 25.0 (IBM Corp., Armonk, NY, USA) with a significance level set at *p* < 0.05.

## 3. Results

### 3.1. Association Between Physical Activity Intensity at Light HR (Resting HR–50% of Maximum HR) and Light/Deep Sleep, Adjusted for Age, Sex, and BMI

Table 2 presents the associations between light-intensity PA (from resting HR to 50% of maximum HR) and sleep outcomes, adjusted for age, sex, and BMI. Higher levels of light-intensity PA were significantly associated with less light sleep (β = −0.042; standard error (SE) = 0.017; *p* = 0.015) and more deep sleep (β = 0.022; SE = 0.010; *p* = 0.023).

### 3.2. Association Between Physical Activity Intensity at Moderate HR (50–70% of Maximum HR) and Light/Deep Sleep, Adjusted for Age, Sex, and BMI

Table 3 shows the results for moderate-intensity PA. No statistically significant associations were found with either light sleep (β = −0.089; SE = 0.052; *p* = 0.090) or deep sleep (β = −0.027; SE = 0.029; *p* = 0.366).

### 3.3. Association Between Physical Activity Intensity at Vigorous HR (70–85% of Maximum HR) and Light/Deep Sleep, Adjusted for Age, Sex, and BMI

Table 4 displays the associations for vigorous-intensity PA. While no significant association was found with light sleep (β = −0.407; SE = 0.322; *p* = 0.206), vigorous PA was positively associated with deep sleep (β = 0.302; SE = 0.038; *p* < 0.001).

## 4. Discussion

The findings of the present study reveal both similarities and differences in the relationships between PA at various intensities and light/deep sleep among pre-adolescents and adolescents from southern Spain. Contrary to initial hypotheses, our results indicate that longer average daily durations of light-intensity PA are associated with reduced light sleep and increased deep sleep. Furthermore, higher levels of vigorous PA were also positively associated with deep sleep. No significant associations were found between moderate PA and sleep, although no detrimental effects of moderate PA on sleep were observed either. Therefore, the findings from prior studies supporting the benefits of moderate-intensity PA remain relevant.

On the one hand, our data show that greater engagement in light-intensity PA is linked to a decrease in light sleep and an increase in deep sleep. This result aligns with the previous literature suggesting that light-intensity activities, such as walking or stretching, help regulate circadian rhythms and promote relaxation, thereby facilitating deeper sleep stages [12,13]. Light PA may also reduce nighttime awakenings and contribute to more consolidated sleep architecture by enhancing parasympathetic nervous system activity, which is known to promote sleep efficiency [13,15].

On the other hand, vigorous-intensity PA (70–85% of maximum HR) was significantly associated with better deep sleep quality. This finding is consistent with prior studies showing that high-intensity activities, such as interval training or competitive sports, enhance slow-wave sleep by increasing homeostatic sleep pressure and reducing sleep onset latency [11,19]. Vigorous PA also raises the core body temperature, and the subsequent post-exercise cooling process may facilitate the transition into deeper sleep stages [12]. In addition, high-intensity exercise has been associated with greater secretion of growth hormone and brain-derived neurotrophic factor (BDNF), both of which play key roles in sleep regulation and neuronal recovery during deep sleep [13,14]. Similarly, Olivo-Martins de Passos et al. [20] found that adolescents who regularly engaged in high-intensity PA showed better sleep outcomes. However, consistent with Gradisar et al. [1], they noted that late-evening exercise may delay sleep onset.

Regarding moderate intensity PA, our analysis did not reveal a significant association with sleep variables. This finding is consistent with that of Sicilia et al. [21], who reported no significant improvements in sleep quality following moderate-intensity exercise. However, most previous studies report that adolescents who meet international PA recommendations (i.e., at least 60 min per day of moderate-to-vigorous intensity activity) tend to exhibit better sleep quality [13,14,15]. A key distinction is that many of these studies group moderate and vigorous intensities into a single category and rely on self-reported questionnaires. In contrast, our use of objective measures allows for a more nuanced analysis of each intensity level independently. Future research should aim to identify the specific types of activities within each intensity range to better understand their distinct effects on sleep and explain why moderate-intensity PA did not yield a significant impact in this study.

### 4.1. Practical Recommendations

Based on the findings of this study, it is recommended that adolescents engage in daily PA, with an emphasis on either extended light-intensity sessions or short bouts of vigorous exercise. Examples include walking throughout the day or participating in recreational sports that involve high-intensity efforts. These contrasting intensities appear to offer the most effective physiological stimulus for improving sleep during pre-adolescence and adolescence. At the family level, it is essential to foster an environment that encourages regular PA, ensuring that adolescents have adequate time and opportunities to engage in both light and vigorous PA, without exceeding levels that may lead to fatigue. Schools should consider implementing integrated and supervised PA programs throughout the school day, as well as promoting extracurricular PA. Educators, particularly Physical Education teachers, can play a critical role by incorporating strategies that increase, motivate, and monitor PA during class sessions. It is also advisable to assess and positively reinforce healthy lifestyle behaviors, such as meeting PA recommendations and maintaining regular sleep routines. Moreover, special attention should be given to adolescents facing sociocultural barriers that may limit their access to or participation in PA. Policymakers should support schools by providing funding and resources to facilitate PA monitoring, for instance, by investing in wearable devices such as smart wristbands to track daily activity and sleep patterns.

### 4.2. Limitations and Strengths

The main limitation of this study is the use of a convenience sample, which limits the generalizability of the findings to the broader adolescent population. In addition, the cross-sectional design prevents the establishment of causal relationships between PA and sleep outcomes. Another limitation concerns the Xiaomi Mi Band 4, which may slightly misclassify data in the light HR zone by overlapping periods of wakefulness and sleep, potentially leading to an estimated overestimation of ~5%. The device may also overestimate total sleep duration and the time spent in light and deep sleep stages, while underestimating wake after sleep onset and the REM phase. Furthermore, the device is not capable of detecting sleep disorders or specific sleep problems. Therefore, caution is advised when interpreting the sleep data, particularly in clinical or diagnostic contexts that require high measurement precision [22]. Future research may benefit from including validated subjective instruments, such as sleep quality questionnaires, to complement objective data. Despite these limitations, the study also presents several notable strengths. It utilized a reliable, validated wearable device and followed a consistent data collection protocol across all participating schools. In addition, the objective classification of PA intensities based on HR zones (light, moderate, and vigorous) offers greater analytical precision. The inclusion of relevant covariates such as age, sex, and BMI also strengthens the internal validity of the findings.

## 5. Conclusions

The aim of the present study was to analyze the associations between daily PA at different intensity levels and light/deep sleep during pre-adolescence and adolescence. The findings indicate that greater daily engagement in light-intensity PA is associated with reduced light sleep and increased deep sleep. Similarly, higher levels of vigorous-intensity PA were also positively related to deep sleep. No negative associations were found between moderate-intensity PA and sleep, suggesting that previously established benefits of moderate PA remain valid. Overall, the results support the promotion of extended light-intensity activities (e.g., walking) and brief bouts of vigorous PA (e.g., sports participation) as effective strategies for improving sleep quality during the pre-adolescent and adolescent stages.

## Figures and Tables

**Table 1:** Sociodemographic characteristics, average daily minutes of physical activity at each intensity level (light, moderate, and vigorous), and time spent in light and deep sleep, segmented by sex.

Variables	All (n = 1072)		Boys (n = 502)		Girls (n = 570)		
	Mean	SD	Mean	SD	Mean	SD	*p*
Age (years)	13.03	1.79	13.06	1.82	13	1.76	0.99
Weight (kg)	51.59	13.6	53.58	15.05	49.59	11.66	<0.001
Height (m)	1.58	0.11	1.60	0.13	1.56	0.086	<0.001
BMI (kg/m^2^)	20.33	4.1	20.49	3.93	20.18	4.08	0.155
Nº of computers at home	2.31	1.4	2.32	1.45	2.29	1.38	0.678
Maternal education level (%)							<0.001
No education	4.9%		2.3%		2.6%		
Primary	10.1%		5.4%		4.7%		
Secondary	13.8%		5.5%		8.2%		
Vocational training	12.7%		6.6%		6.1%		
University	37.3%		16.8%		20.6%		
Unknown	21.2%		13%		8.2%		
Min. of PA/day at light HR	1031.76	215.44	1025.02	230.67	1037.42	201.81	0.317
Min. of PA/day at moderate HR	67.67	54.86	65.43	47.10	69.55	60.59	0.239
Min. of PA/day at vigorous HR	3.52	8.71	3.67	8.95	3.40	8.5	0.620
Sleep variables							
Average of Min./day of Light Sleep	363.40	97.62	364.63	98.85	362.42	96.69	0.717
Average of Min./day of Deep Sleep	97.66	52.55	92.77	50.18	101.57	54.1	0.007

Notes: Data are presented as means for continuous variables and frequencies (%) for categorical variables. BMI = body mass index; SD = standard deviation; PA = physical activity; HR = heart rate.

**Table 2:** Association between light-intensity PA (resting–50% of maximum HR) and light/deep sleep, adjusted for age, sex, and BMI.

Variable	Light Sleep			Deep Sleep		
	β	SE	*p*	β	SE	*p*
Age (years)	−17.72	1.66	<0.001	0.483	0.940	0.607
Sex	−4.45	5.74	0.438	7.59	3.24	0.019
BMI (kg/m^2^)	−0.651	0.751	0.386	−0.496	0.424	0.243
Light-intensity PA (min/day)	−0.042	0.017	0.015	0.022	0.010	0.023

Note: Non-standardized Beta (β), standard error (SE), BMI = body mass index (kg/m^2^).

**Table 3:** Association between moderate-intensity PA (50–70% of maximum HR) and light/deep sleep, adjusted for age, sex and BMI.

Variable	Light Sleep			Deep Sleep		
	β	SE	*p*	β	SE	*p*
Age (years)	−18.86	1.66	<0.001	0.727	0.939	0.439
Sex	−3.59	5.75	0.532	7.384	3.24	0.023
BMI (kg/m^2^)	−0.485	0.759	0.523	−0.435	0.429	0.311
Moderate-intensity PA (min/day)	−0.089	0.052	0.090	−0.027	0.029	0.366

Note: Non-standardized Beta (β), standard error (SE), BMI = body mass index (kg/m^2^).

**Table 4:** Association between vigorous-intensity PA (70–85% of maximum HR) and light/deep sleep, adjusted for age, sex, and BMI.

Variable	Light Sleep			Deep Sleep		
	β	SE	*p*	β	SE	*p*
Age (years)	−18.05	1.63	<0.001	0.875	0.903	0.333
Sex	−5.42	5.72	0.343	6.39	3.16	0.044
BMI (kg/m^2^)	−0.462	0.751	0.539	−0.502	0.414	0.226
Vigorous-intensity PA (min/day)	−0.407	0.322	0.206	0.302	0.038	<0.001

Note: Non-standardized Beta (β), standard error (SE), BMI = body mass index (kg/m^2^).

## Data Availability

The original contributions presented in the study are included in the article, further inquiries can be directed to the corresponding author.
